# The Angiotensin II Type 2 Receptor, a Target for Protection and Regeneration of the Peripheral Nervous System?

**DOI:** 10.3390/ph14030175

**Published:** 2021-02-24

**Authors:** Aurore Danigo, Amandine Rovini, Flavien Bessaguet, Hichem Bouchenaki, Amandine Bernard, Franck Sturtz, Sylvie Bourthoumieu, Alexis Desmoulière, Laurent Magy, Claire Demiot

**Affiliations:** 1 EA 6309—Myelin Maintenance & Peripheral Neuropathy, Faculties of Medicine and Pharmacy, University of Limoges MMNP, 87000 Limoges, France; flavien.bessaguet@etu.unilim.fr (F.B.); hichem.bouchenaki@unilim.fr (H.B.); amandine.bernard@unilim.fr (A.B.); franck.sturtz@unilim.fr (F.S.); sylvie.bourthoumieu@unilim.fr (S.B.); alexis.desmouliere@unilim.fr (A.D.); laurent.magy@unilim.fr (L.M.); claire.demiot@unilim.fr (C.D.); 2 BioTis, INSERM U1026, University of Bordeaux, 33000 Bordeaux, France; amandine.rovini@u-bordeaux.fr; 3 Department of Neurology, Reference Center for Rare Peripheral Neuropathies, University Hospital of Limoges, 87000 Limoges, France

**Keywords:** angiotensin II type 2 receptor, peripheral nervous system, neuroregeneration, neuroprotection, pain

## Abstract

Preclinical evidence, accumulated over the past decade, indicates that the angiotensin II type 2 receptor (AT2R) stimulation exerts significant neuroprotective effects in various animal models of neuronal injury, notably in the central nervous system. While the atypical G protein-coupled receptor superfamily nature of AT2R and its related signaling are still under investigation, pharmacological studies have shown that stimulation of AT2R leads to neuritogenesis in vitro and in vivo. In this review, we focus on the potential neuroprotective and neuroregenerative roles of AT2R specifically in the peripheral nervous system (PNS). The first section describes the evidence for AT2R expression in the PNS and highlights current controversies concerning the cellular distribution of the receptor. The second section focuses on AT2R signaling implicated in neuronal survival and in neurite outgrowth. The following sections review the relatively few preclinical studies highlighting the putative neuroprotective and neuroregenerative effects of AT2R stimulation in the context of peripheral neuropathy.

## 1. Introduction

There is a large unmet clinical need for novel therapeutic approaches to reduce disabilities (sensory impairment, motor deficit) and to improve overall quality of life in patients with peripheral neuropathies. The development of effective therapeutic solutions is hampered by the vast etiological spectrum of underlying causes and the persistent gaps in our understanding of the pathophysiological processes involved in peripheral neuropathy. However, accumulating evidence suggests a significant contribution of the renin-angiotensin system (RAS) in both neuroprotection and neuroregeneration.

**Figure 1:**
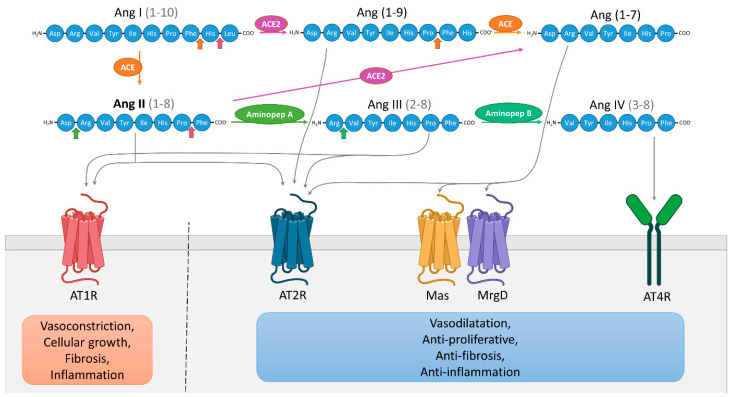
Schematic representation of the renin-angiotensin system (RAS). Angiotensin (Ang) I is cleaved by angiotensin-converting enzyme (ACE) to Ang II which can be then cleaved to Ang III by aminopeptidase A, then further cleaved to Ang IV by aminopeptidase B. Ang I can also be cleaved by ACE2 to produce Ang (1–9) which can be cleaved into Ang (1–7) by ACE. Ang (1–7) can be directly generated from Ang II by ACE2. Ang (1–7) binds and activates the receptors Mas and Mas-related G protein-coupled receptor member D (MrgD), Ang IV binds to AT4R, Ang III activates AT1R and AT2R and Ang (1–9) directly activates AT2R. Functionally, it is possible to simplify the RAS into two distinctive pathways [12]. The first involves over-activation of AT1R by Ang II/Ang III, promoting cellular growth, vasoconstriction, fibrosis and inflammation. The second involves the interactions between AT2R and Ang II/Ang (1–9)/Ang III, but also Ang (1–7) and Mas/MrgD receptors, and Ang IV and AT4R, leading to vasodilatation and anti-proliferative, anti-fibrotic, anti-inflammatory effects.

The RAS is well-described and known to regulate arterial blood pressure and ionic homeostasis [1]. The sequential enzymatic cascade of RAS is initiated with renin, a catalytic enzyme produced by kidney and secreted into the systemic circulation, which cleaves liver-derived angiotensinogen (AGT) to produce the decapeptide angiotensin I (Ang I). Pulmonary angiotensin converting enzyme (ACE) converts Ang I into angiotensin II (Ang II), the main active component of the RAS. Ang II binds with high and similar affinity to its two principal receptors in humans, the Ang II type 1 receptor (AT1R) and the Ang II type 2 receptor (AT2R) [2]. Other angiotensinogen-derived peptides have been described and are shown in Figure 1. The RAS was first described as an endocrine system. However, it is now considered to be a “ubiquitous” system that is expressed locally in many tissues exerting multiple autocrine/paracrine effects with implications in tissue physiology and homeostasis. The first demonstration of Ang II presence in tissues, in the arterial wall of sheep, dates back to 1980 [3]. Subsequent studies have quantified the synthesis of Ang II by the use of radiolabeled ligands in the heart, kidneys and adrenal glands [4,5,6,7]. Additionally, components of a local RAS have been detected in several tissues including skin, bone, adipose tissue and inflammatory cells [8,9,10]. The localization and effects of local RAS are described in further detail elsewhere [11].

AT1R and AT2R are both seven-transmembrane receptors displaying a similar affinity to Ang II although these two receptors differ in their amino acid sequence, tissue-specific expression and functional effects. The main role of AT2R is to inhibit actions mediated by AT1R by decreasing cell growth and proliferation while promoting cell differentiation, in addition to a vasodilatory action and a decrease in blood pressure [12]. As demonstrated in several studies, AT2R expression is temporally regulated. Ligand binding, in situ hybridization and autoradiography studies show that AT2R is widely expressed during fetal life, whereas its expression is maintained at low levels in all organs in adults, contrary to AT1R which is preferentially expressed in adults [13,14]. On the other hand, recent western blot and RT-PCR studies show that AT2R shows significantly higher expression in adult than in fetal and neonatal rodent tissues, with the exception of skin [15,16]. Such a discrepancy challenges the role of AT2R during development. Moreover, inverse expression profiles of AT1R and AT2R during development have been reported, with decreased expression of AT1R and an increased expression of AT2R in the adult versus fetal stage, suggesting a crucial interacting role between the two receptors [16]. Moreover, AT1R/AT2R heterodimerization has previously been shown in non-neuronal cell types, illustrating the strong link between these two receptors [17]. AT2R expression is also dramatically increased in tissue under pathological conditions, for example during nerve crush injury or inflammation, suggesting a possible role of AT2R in tissue repair and more particularly in neuroregeneration [18,19,20].

While numerous studies have focused on protective and regenerative properties of AT2R in the central nervous system [21,22,23], very few have considered a role for AT2R in the peripheral nervous system (PNS). Several reports have emphasized the role of peripheral AT2R in the modulation of pain [24,25,26,27,28,29,30]. However, the potential effects of Ang II/AT2R in neuroprotection/neuroregeneration in the PNS have been understudied and remains poorly understood. A few pharmacological studies, some from our team, highlight the beneficial effect of AT2R stimulation in a rodent model of traumatic- and drug-induced peripheral neuropathy [19,31,32,33,34]. Here, we aim to review the current knowledge on the role of AT2R and the therapeutic effect of respective agonists or antagonists in the treatment of various types of peripheral neuropathies. Basic information concerning the AT2R and its distribution within the PNS are introduced first.

## 2. Evidence for AT2R Expression in the Peripheral Nervous System

Over the past fifteen years, many studies have supported the concept of a local RAS and its potential role in the PNS, and particularly in the sensory nervous system [27,35,36,37,38]. Nevertheless, the distribution and expression level of AT2R in the PNS has been subject of controversy.

**Figure 2:**
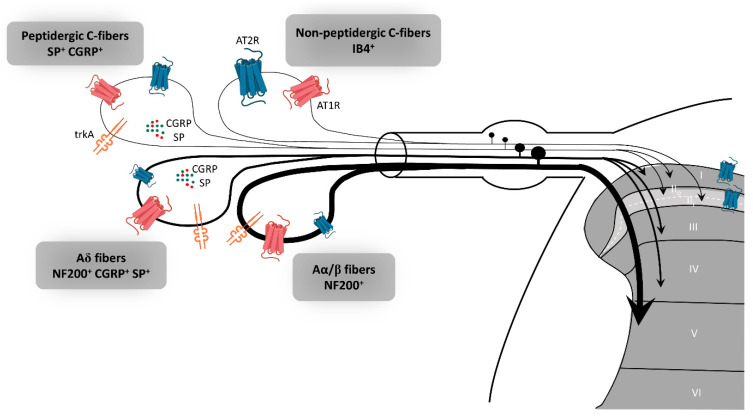
Expression of AT2R in the sensory peripheral nervous system. According to the most recent immunohistochemistry study on the expression of AT2R in DRG and in the spinal cord of rat, AT2R would be expressed by almost all types of sensory neurons, though to different degrees [18]. Non-peptidergic C-nociceptors expressing IB4 are strongly stained for AT2R. Some small and medium DRG neurons co-expressed AT2R and trkA, a marker of nociceptive neurons (peptidergic C and Aδ neurons). The few large neurons which express AT2R are stained for NF200 and trkA, markers of Aα/β nociceptive neurons. Most ATR2^+^ neurons are also AT1R^+^. AT1R: angiotensin II type 1 receptor, AT2R: angiotensin II type 2 receptor, CGRP: calcitonin gene-related peptide, DRG: dorsal root ganglion, IB4: isolectin 4, NF200: neurofilament 200, SP: substance P, trkA: tropomyosin receptor kinase type A.

In rat dorsal root ganglion (DRG), AT2R expression, at the mRNA and protein levels, is tightly regulated throughout development to switch later to a restricted subpopulation of C-nociceptor neurons during adult life [39]. Recently, preferential expression of AT2R in non-peptidergic isolectin B4 (IB4^+^) C-nociceptor neurons was confirmed in adult rat DRG neurons [18]. The authors showed that AT2R is also expressed by some peptidergic small C- and medium Aδ-neurons as well as by a few large Aα- and Aβ-neurons, and that almost all AT2R^+^ DRG neurons co-expressed AT1R [18] (Figure 2). In humans, positive AT2R-immunolabelling was also shown in small-diameter DRG neurons and in nerve endings in the sub-epidermis and dermis, in urinary bladder, in vestibule and in the myenteric plexus [27,38,40]. This expression profile of AT2R in the PNS suggests its involvement in the development of sensory and nociceptive functions. However, immunohistochemistry (IHC)-based results must be viewed with caution due to the poor specificity of commercially-available AT2R antibodies [24,40]. One study has demonstrated the specificity of one commercial AT2R antibody, ab19134 (Abcam), using AT2R-expressing HEK-cells vs. non-transfected HEK-cells [41]. Nevertheless, recently, a research group reported no difference in AT2R signal intensity in DRG sections from wild type- and *agtr2* (the AT2R gene) KO-mice using these same AT2R antibodies [24].

At the mRNA level, AT2R is upregulated in pathological conditions in the PNS, as has been shown in other tissues such as infarcted heart and regenerating skeletal muscle [42,43]. In response to nerve axotomy and crush injury, AT2R mRNA levels are strongly increased in adult DRG and sciatic nerve fibers in rats [44]. A role for AT2R in Schwann cell (SC)-mediated healing actions has been evoked since the kinetics of AT2R mRNA expression is closely linked to the kinetics of SCs differentiation during nerve recovery. It is important to note that quantification of total AT2R mRNA from DRG encompasses different cell types (i.e., sensory neurons, satellite cells, immune cells, and SCs). SCs themselves have been shown to express AT1R and AT2R in vitro and in fresh samples of rat sciatic nerve, with a higher proportion of AT2R than AT1R [45].

Recently, Shepherd et al. demonstrated that mouse and human DRG neurons do not express AT2R [24]. By combining different technical approaches to investigate the expression of AT2R, the authors pointed out the lack of AT2R expression in mouse DRG neurons, either at the protein- or mRNA-level. In line with this, Ang II did not induce either calcium influx and electrophysiological responses or intracellular signaling in cultured primary mouse DRG neuron. In addition, a lack of GFP signal was observed in DRG sections from AT2R-EGFP reporter mice, suggesting that *agtr2* was not expressed either in neurons or in non-neuronal cells of mouse DRG. However, they revealed that peripheral macrophages express a functional AT2R and these could be implicated in Ang II-induced peripheral mechanical pain sensitization [24]. One could envision a paracrine system between DRG neurons that synthesize Ang II and non-neuronal AT2R^+^ cells such as SCs and/or immune cells that are recruited only during nerve injury [46]. In this respect, the CD3^+^ T-cells which are involved in mechanical pain in response to chronic constriction injury (CCI) [41] might express AT2R. This cell-cell dialogue, mediated by AT2R in the PNS, could play a key role in neuroprotective and neuroregenerative processes.

With regard to the expression of AT2R by DRG neurons, it appears that species differences likely exist. Most studies conducted in rats have found AT2R to be expressed in DRG neurons while studies performed in mouse DRG neurons have given opposite results [24]. One explanation might be that two isoforms of AT2R exist in mice, as is the case for AT1R (AT1a and AT1b) in mice and rats, and as has already been suggested in the rat brain [47]. In this respect, AT2R-antibodies could detect another isoform of AT2R, which could be expressed in DRG neurons of AT2R KO-mice. It is also important to note that AT2R-deficient mice often only have part of the receptor affected, thus potentially allowing the expression of a truncated form of the protein, which is possibly detected by some antibodies [48,49]. Another hypothesis is that AT2R mRNA is synthesized in DRG neurons under pathological conditions (lesion, crush, section) and transported along microtubules to nerve terminals where it is then translated by a local system [50]. This would provide a rationale for the discrepancies in results obtained from IHC on cultured DRG and DRG sections. Further studies are required to elucidate the questions concerning AT2R expression in the PNS and to understand their function.

## 3. AT2R Signaling

**Figure 3:**
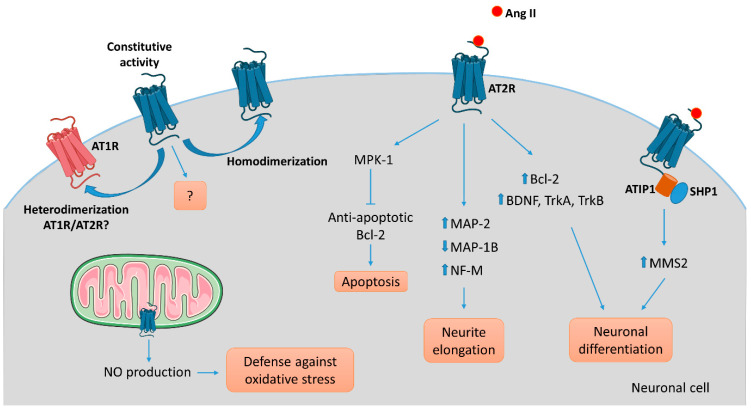
AT2R-mediated intracellular signaling pathways involved in neuronal cells. Several intracellular signaling pathways are associated with AT2R activation in neuronal cells leading to apoptosis via activation of the phosphatase MPK-1, neurite elongation via reorganization of the cytoskeleton, or neuronal differentiation in part via upregulation of growth-factors, depending on the cell type, the context, and the environment. To date, ATIP1 is identified as a partner of AT2R involved in intracellular signaling pathway and trafficking in neuronal cells. While the evidence of an ATIP/AT2R complex comes from experiments on central nervous system neurons, we could hypothesize that a similar system exists in PNS neurons. AT2R signaling may antagonize AT1R-mediated signaling by a direct heterodimerization between the two. As well, homodimerization of AT2R might result in ligand-independent signaling.

The AT2R gene was cloned in the early 90′s and the receptor has been attributed numerous functions. However, its signaling pathway remains difficult to elucidate [51,52]. AT2R belongs to the G protein-coupled receptor superfamily (GPCR). However, aside from signaling through G-protein-dependent mechanisms, activation of this receptor by G protein-independent intracellular signaling in neurons makes it an “atypical” or “non-canonical” GPCR. The recently described crystal structure of human AT2R bound to either Ang II or an AT2R-selective ligand, allowed the receptor to be captured in an active-like conformation and provided structural insights into a moderate coupling to G proteins [53,54]. Continuing efforts investigating the conformational arrangement of this receptor are necessary to complete our understanding of AT2R activation and signaling and will aid in better design of targeted compounds [55]. In the following section, we will specifically review the role of AT2R signaling implicate in neuronal survival and neurite outgrowth and summarize the current findings in a schematic (Figure 3).

To understand AT2R signal transduction, several cellular tools have been used. Among them, the rat pheochromocytoma PC12W cell line, of neuronal origin, has been particularly useful since these cells mostly express AT2R rather than AT1R. Several studies, focused on the effect of Ang II on PC12W cells, have demonstrated that AT2R mediates programmed cell death through inactivation of mitogen-activated protein kinase (MAPK) inhibition of the anti-apoptotic Bcl-2 protein resulting eventually in induction of apoptosis [56,57]. This apoptotic function of AT2R was once hypothesized to be involved in developmental biology and pathophysiology. However, it was later demonstrated that AT2R stimulation by C21, a specific agonist [58], induced RNA expression of Bcl-2 and increased the level of neurotrophins (BDNF, TrkA and TrkB) in vitro in primary neurons and in vivo in a model of spinal cord injury [59]. In quiescent PC12W cells, AT2R stimulation by Ang II treatment leads to neurite formation [60]. In this case, the signaling involves an increase in polymerized β-tubulin, upregulation of microtubule-associated protein (MAP)-2 and down-regulation of MAP-1B levels. Similar observations were made in PC12W cells differentiated by nerve growth factor (NGF) and in undifferentiated NG108-15 cells (mouse neuroblastoma x rat glioma hybrid cell line) [60,61]. MAP-2 proteins are known to interact with microtubules, neurofilaments and actin, and contribute to the maintenance of the neuronal cytoskeleton. Similarly, MAP-1B regulates branching and neurite direction during DRG neuron regeneration [62]. A significant decrease in MAP-2 expression was observed in DRG following CCI to the sciatic nerve in rats, suggesting the involvement of MAP-2 in the early response to nerve injury [63]. Ang II treatment also diminishes the expression of neurofilament-M at the protein and mRNA levels in PC12W cells, and this effect is suppressed by preventive treatment with PD123177 (an AT2R antagonist) [64]. Thus, AT2R stimulation in vitro decreases proliferation and leads to neuronal differentiation and to anarchic neurite elongation via reorganization of cytoskeletal components. In undifferentiated NG108-15 and PC12W cells, both cell types expressing AT2R but not AT1R, Ang II-induced neuronal differentiation, and thus neurite elongation, is counteracted by AT1R stimulation in differentiated neuronal cells expressing AT1R [61]. Therefore, these data highlight the precise regulation existing between AT2R and AT1R during neuronal differentiation and neurite elongation. In NG108-15 cells, Ang II/AT2R interaction leads to neurite outgrowth through a sustained activation of p42/p44 MAPK and phosphorylation of trkA [65,66]. The link between AT2R and activation of p42/p44 MAPK leading to neurite outgrowth was further confirmed in cultured adult rat DRG neurons, another optimal in vitro model in which to investigate the effects of AT2R modulation on PNS neurons [67]. In this later study, neurons treated with Ang II presented denser and much longer neurites than controls, confirming results obtained in neuronal cell lines.

It has also been demonstrated that the morphological differentiation induced by Ang II/AT2R involves an increase in nitric oxide (NO) production in the NG108-05 cell line [68]. An increase of neuronal NO synthase (nNOS) was observed in cultures of rat DRG neurons under stress conditions [69]. The neuroprotective role of NO has already been described in vitro and in vivo in rat DRG neurons [69,70,71]. In vivo, inhibition of nNOS aggravates DRG neuron injuries, such as in a rat model of sciatic nerve transection, suggesting a neuroprotective role of NO [71]. A more detailed review of the role of NO in neuronal proliferation, survival and differentiation can be found elsewhere [72]. A functional mitochondrial angiotensin system has been reported in several cellular types, including neuronal cells [73]. AT2R was localized on the inner mitochondrial membrane by immunogold electron microscopy, and its stimulation increased NO production, thus decreasing mitochondrial respiration [74]. This phenomenon could represent a defense against oxidative stress, and a neuroprotective function of AT2R. However, the presence of AT2R in mitochondria from PNS neurons has not thus far been investigated.

In 2007, Li et al., showed, in neuronal cells, that AT2R interacts with ATIP1, the first member of the AT2-interacting protein (ATIP) family, to induce neuronal differentiation via upregulation of methane methylsulfonate-sensitive 2 (MMS2) [75]. ATIPs are a family of proteins encoded by alternative splicing of a single gene called *mtus1* (Microtubule associated tumor suppressor 1); ATIP1, ATIP3 and ATIP4 being the major isoforms. ATIP1 and ATIP3 are widely distributed whereas ATIP4 is restricted to the central nervous system [76]. The ATIPs are cytosolic proteins that constitutively interact with the C-terminal domain of AT2R and are involved in intracellular transport and signaling pathways of AT2R, depending on the cell-type and on the isoform [77,78]. ATBP50, the murine ATIP1, was identified as a Golgi-associated protein involved in the transport of the AT2R to the cell membrane [78]. In neuronal cells, AT2R activation induces the ATIP1-Src homology phosphatase 1 (SHP1) complex, leading to transcriptional activation of the DNA repair enzyme MMS2, and then induction of neuronal differentiation [75].

While progress has been made in understanding AT2R signaling, this receptor remains an enigma. Other review articles have emphasized in great detail the limitations of AT2R signaling studies [12,79,80,81].

## 4. Neuroprotection

Neuroprotection is defined as the ability for a therapy to prevent neuronal cell death by intervening in and inhibiting the pathogenic cascade that results in cell dysfunction and eventually in neuronal death. Most peripheral neuropathies are axon length dependent and result in distal axonal degeneration rather than loss of neuronal cell bodies. Thus, the ideal neuroprotective molecule should limit distal axonal degeneration, favor axon-glia interactions, and promote axonal regeneration and connection with the target cells. The antioxidant and anti-inflammatory axes are key mechanisms involved in neuroprotection.

In the central nervous system, blockade of AT1R protects against ischemic brain damage, notably by reversing oxidative stress and inflammation [82,83]. These neuroprotective effects during AT1R blocker treatment are associated with a relative increase of AT2R stimulation by Ang II [84]. Further studies evaluating direct AT2R stimulation by the agonists CGP42112 or C21 have confirmed anti-inflammatory and tissue repair effects of AT2R in rodent models of stroke using middle cerebral artery occlusion [85,86,87]. Protective effects of AT2R modulation in diverse neuronal populations in a wide range of brain injuries including ischemic strokes, ischemia/reperfusion injury, inflammation and others, have already been reviewed elsewhere [88,89].

A small number of in vivo preclinical studies have highlighted the putative neuroprotective effect of AT2R stimulation on peripheral neuropathies. In the first study, candesartan (an AT1R antagonist) treatment prevented functional sensory neuropathy in a rat model of type 2 diabetes of fructose-induced insulin resistance [32]. In this model, the peripheral neuropathy was characterized by hypoalgesia in response to a noxious thermal stimulus. These authors also showed that insulin resistance resulted in a decrease of calcitonin gene-related peptide (CGRP)-immunoreactive nerves in mesenteric arteries and an increase of AT2R expression and dysfunction in DRG neurons. This significant increase of AT2R expression in the DRG of fructose drinking rats (FDR) reinforces the concept that AT2R plays an important role in nerves under pathological conditions. When candesartan was administered at the same time as fructose in FDR, sensory nerve disorders were improved, the density of CGRP^+^ nerves was increased to match control levels and the high AT2R expression in DRG neurons was suppressed. Moreover, in FDR, AT1R blockade improved AT2R-mediated neurite outgrowth and restored Akt signaling [32]. Once again, these results reveal the strong functional interconnection that is present between AT1R and AT2R in peripheral nerves.

In our previous studies, we have shown several lines of evidence that direct or indirect stimulation of AT2R could prevent the development of sensory impairment in mouse models of toxin-induced neuropathies. First, in a mouse model of resiniferatoxin (RTX)-induced functional neuropathy, we showed that AT1R blockade by preventive administration of candesartan restored the RTX-induced thermal hypoalgesia and improved the depletion of substance P (SP) and CGRP in intraepidermal nerve fibers (IENFs) and in DRG neurons. This effect of candesartan was blocked by PD123319 (or EMA200), an AT2R blocker, and also in the AT2R KO mouse model. Therefore, we concluded that the neuroprotective effect of AT1R blockade was mediated by the promotion of AT2R activation by Ang II and the increase in tissue Ang II level [31]. Secondly, in a mouse model of vincristine (VCR)-induced tactile allodynia, administration of candesartan and C21 accelerated the recovery of normal tactile sensitivity. This beneficial effect of candesartan and C21 was abolished by PD123319. Both drugs also prevented VCR-induced non-peptidergic IENF loss [19]. We also showed that direct AT2R stimulation by C21 prevented myelinated fiber loss and the enlargement of myelinated axonal diameter induced by VCR in our mouse model [19].

Interestingly, all of the above studies which highlight the neuroprotective effect of AT2R in the PNS were performed using rodent models of “toxin”-induced neuropathy, rather than traumatic models.

## 5. Neuroregeneration

In contrast to the central nervous system, injured axons in the PNS maintain regenerative capacities. Neuroregeneration involves neuronal and non-neuronal responses resulting in restoration of neuronal connectivity and functional recovery. Injured peripheral axons have to regrow over long distances to reestablish synaptic connections with their targets in the periphery. Failure of axonal regrowth contributes to muscle atrophy or to loss of function of end-organs. In order to combat these effects and improve functional outcomes following PNS injury, the goal of an ideal treatment would be to accelerate axonal regeneration.

### 5.1. In Vitro

The neuroregenerative properties of AT2R stimulation were first highlighted in the PC12W neuronal cell line, which is responsive to NGF treatment. Data in PC12W cells showed that AT2R stimulation by Ang II enhances neurite elongation and promotes NGF-induced neuronal differentiation. AT2R stimulation inhibits growth factor-induced proliferation and enhances NGF-mediated growth arrest. All of the effects of Ang II in PC12W and NG108-15 cells were suppressed by pretreatment with PD123319 [90,91]. The same neuroregenerative effect of Ang II was observed in primary cultures of neonatal rat DRG neurons. This Ang II effect is inhibited by PD123177 (an AT2R blocker) treatment but not by losartan (an AT1R blocker) [20]. Using the same experimental paradigm, others have recently shown that the neuritogenesis properties of Ang II are counteracted by AT2R (PD123312)- as well as by AT1R (azilsartan)- antagonism [18]. Moreover, addition of Ang II in the culture medium induced neurite outgrowth in small to medium sized neurons positive for peripherin and this neuritogenesis was inhibited by PD123319 [37]. Hashikawa-Hobara and Hashikawa (2016) showed that AT2R-stimulation by CGP42112, a selective agonist of AT2R, induced neurite outgrowth in a primary culture of adult mouse DRG cells [92]. This result was also previously reported using rat and human DRG cell cultures treated with C21 or Ang II [27,67]. This positive effect of AT2R stimulation on neurite length and neurite density, shown with Gap43 staining (an axonal regeneration marker), was reduced after EMA401 (an AT2R antagonist) treatment [67].

### 5.2. In Vivo

To explore the neuroregenerative effects of drugs on the PNS, nerve transection and crush injury models in rodents have been widely used as the most representative models of traumatic peripheral neuropathy allowing the identification of key repair mechanisms. Local administration of Ang II in a rat model of sciatic nerve crush showed enhanced neuroregenerative mechanisms by accelerating recovery of sensorimotor function and by enhancing axonal regeneration and myelination [34]. All of these Ang II-mediated regenerating effects were blocked by PD123319. The authors concluded that this AT2R-dependent neuroregenerative effect could be due to the stimulation of AT2R in SCs with subsequent nuclear translocation of NF-κb, which is essential for myelin formation. In a rat model of inflammatory pain, one study has reported that hind paw injection of complete Freund’s adjuvant (CFA) induced cutaneous hyper-innervation associated with mechanical and thermal hypersensitivity at 3 days post-CFA injection. Simultaneous infusion of an AT2R blocker (PD123319) with CFA abolished dermal and epidermal hyper-innervation and reduced associated-hypersensitivity [93,94]. These results highlight the involvement of AT2R activation in inflammation-induced cutaneous hyper-innervation. The authors also demonstrated that a local RAS is expressed by the inflammatory cells, which have been recruited at the CFA injection site. Further investigations were performed on tissue from patients with provoked vulvodynia (PVD) [38]. PVD is characterized as a localized provoked pain in the vulvar vestibule, attributed to an increase of nociceptors in the vestibular endoderm and elevated numbers of inflammatory cells [95,96,97]. Tender tissue from PVD patient shows hyper-innervation and an increased number of T-cells, B-cells and macrophages compared with non-tender areas. The authors demonstrated that all of the necessary RAS components to synthesize Ang II are present in tender tissue and provided by inflammatory cell migration and/or proliferation. They also showed that neurite outgrowth is induced by tender tissue-conditioned medium in primary cultures of neonatal rat DRG neurons, and that this effect is prevented by adding Ang II antibody or PD123319 [38]. Recently, Benitez et al. 2020 showed that pharmacological inhibition of AT1R has greater impact on CGRP^+^ axonal endings whereas inhibition of AT2R has more effect on IB4^+^ nociceptors under physiological conditions [18]. They reported that AT2R but also AT1R are involved in neuritogenesis of these nociceptors. These data confirm, in vivo, both the neuroregenerative properties of AT2R stimulation in the PNS and the interplay of AT1R and AT2R.

## 6. Concluding Remarks

Recent data have provided evidence that AT2R plays an important role in the protection and regeneration of the sensory nervous system. The complex and enigmatic nature of AT2R, as coined by Sadybekov and Katritch, and its related signaling, leaves many unanswered questions about its function [55]. Intracellular events generated by what is considered as an atypical GPCR are numerous and tissue-type dependent. A better understanding of AT2R ligand-independent activity and signaling cascades is critical and would enable us to define the physiological and functional contributions of this receptor.

Despite controversies about the expression of AT2R in the PNS, mainly due to the poorly characterized antibodies that are commercially available, pharmacological studies have shown that stimulation of AT2R leads to neuritogenesis in vitro and in vivo (Table 1), supporting the rationale that the peripheral AT2R may be an effective therapeutic target. In this instance, development of AT2R agonists could be beneficial in the case of traumatic or toxic peripheral neuropathies involving sensory axon loss. On the other hand, AT2R agonists may be deleterious in the case of inflammatory disease involving hyper-innervation. Thus, in vestibulodynia, treatment with PD123319 (an AT2R blocker) appears to be effective in reducing AT2R-dependent hyper-innervation and associated pain.

However, the relevance of AT2R targeting remains to be fully established since the latest clinical trials with EMA401 performed by Novartis have been terminated prematurely due to safety issues [28]. A similar contradiction applies to our understanding of AT2R involvement in modulation of pain [98]. While some studies have reported an analgesic effect following AT2R blockade, others have shown that AT2R stimulation prevents neuropathic pain [98]. Improved knowledge of the RAS and particularly of AT2Rs in the PNS provide possible new approaches, novel methods and promising targets to address unmet medical needs among patients with peripheral neuropathy.

## Figures and Tables

**Table 1:** Neuroprotective and neuroregenerative effect of AT2R stimulation in the peripheral nervous system.

Model	Species	Effect of Neuropathy	Treatment	Administration	Effect of Treatment	Antagonism	AT2R Expression	Ref.
**Topical phenol treatment**	Wistar rat (6 week-old)	Decrease of CGRP nerve fibers in mesenteric arteries	Losartan + Ang II	Osmotic pump	Increase of CGRP nerve fibers	Effect suppressed by PD123319	DRG neuron (WB)	[33]
**RTX-induced sensory neuropathy**	Male Swiss mouse (25–30 g)	Depletion of CGRP and SP in IENFs and in DRG neurons	Candesartan	I.P..	Improvement of the neuropeptide depletion	Effect counteracted by PD123319 and in AT2R-KO mice	/	[31]
**Vincristine-induced neuropathy**	Male Swiss mouse (25–30 g)	Nonpeptidergic IENFs loss, loss of myelinated fibers and enlargement of axonal diameter in sciatic nerve	Candesartan and C21	I.P.	Prevention of IENF loss by candesartan and C21. Prevention of myelinated nerve fiber impairment by C21	Effects counteracted by PD123319	/	[19]
**Sciatic nerve crush**	Male Sprague-Dawley rats (200–250 g)	Axonal degeneration and demyelination	Ang II	Osmotic pump	Enhancement of axonal regeneration and myelination	Effect lost with PD123319	SCs	[34]
**Hind paw inflammation (CFA injection)**	Female Sprague-Dawley rats (190–200 g)	Increase of PGP9.5^+^ nerve fibers in dermis and epidermis. Increase of CGRP^+^ nerve fibers in dermis	PD123319	Osmotic pump	Decrease of CFA-induced hyper-innervation in dermis and epidermis.	/	/	[93]
**Rat DRG cells**	Adult female Wistar rat	/	Ang II	/	Ang II increased neurite length and density	Effects counteracted by EMA401	DRG neurons (ICC, IHC)	[67]
**Avulsed Human cervical DRG cells**	From surgical nerve repair procedure	/	EMA401	/	Mean neurite density was reduced by EMA401	/	DRG cells	[67]
**Adult mouse DRG cells**	Male C57BL6J mice (25–30 g)	/	NGF + PD123319 or AT2R siRNA	/	AT2R inhibition is associated with decrease of NGF-induced neurite outgrowth	/	DRG cells	[92]

Ang II: angiotensin II, CFA: complete Freund’s adjuvant, CGRP, calcitonin gene-related peptide, DRG: dorsal root ganglia, IENF: intraepidermal nerve fiber, ICC: immunocytochemistry, IHC: immunohistochemistry, I.P: intra-peritoneal, NGF: nerve growth factor, PGP9.5: protein gene product 9.5, RTX: resiniferatoxin, SCs: Schwann cells, SP: substance P, WB: western blot.

## Data Availability

No new data were created or analyzed in this study. Data sharing is not applicable to this article.
